# Diagnostics of CAPS in Quebec thanks to teaching program

**DOI:** 10.1186/1546-0096-13-S1-P35

**Published:** 2015-09-28

**Authors:** A-L Chetaille, A Albert, K Adams, P Fortin, L Michou

**Affiliations:** 1 CHU de Québec-Université Laval, Rhumatologie pédiatrique, Québec (Québec), Canada; 2 CHU de Québec, Rhumatologie, Québec (Québec), Canada

## Introduction

We describe here the first 16 patients diagnosed with Cryopyrin-Associated Periodic Syndromes (CAPS) in Quebec for the past ten years. These disorders are unfortunately often left undiagnosed and untreated because of lack of knowledge.

## Objectives

To inform the medical community on these diagnosis to avoid misdiagnoses and to reduce the time to reference to the appropriate specialist.

## Methods

We gave lectures on an auto-inflammatory learning program dedicated to medical care practitioners from different specialities to educate them on these diagnosis. This program was given to pediatricians, adult rheumatologists, allergists, dermatologists, infectious disease specialists and internists.

## Results

16 patients were diagnosed with CAPS. The adult and pediatric rheumatologist who developped and gave the learning program diagnosed 11 cases, directly or referred by allergists, pediatricians and dermatologists. 4 adult rheumatologists diagnosed one or two cases of CAPS each. 8 diagnostics were made at pediatric ages, 8 at adult ages. 2 presented as chronic infantile neurological cutaneous and articular syndrome (CINCA), 9 as Muckle-Wells syndrome (MWS), and 5 as familial cold autoinflammatory syndrome (FCAS). Diagnosis was supported in 7 cases by genetic mutation of NLRP3 (44%). Delay in diagnosis was between 2 weeks and 50 years, mean 15,8 years.

## Conclusion

Developping teaching tools permitted to educate physicians on these underdiagnosed diseases. This population of 16 CAPS represents the first cases diagnosed in Quebec thanks to interaction between specialities. Improvement can still be made to reduce time to diagnosis.

